# Updated distribution and host records for the argasid tick *Ornithodoros* (*Pavlovskyella*) *zumpti*: A potential vector of African swine fever virus in South Africa

**DOI:** 10.4102/ojvr.v88i1.1960

**Published:** 2021-12-09

**Authors:** Anthony F. Craig, Livio Heath, Jan E. Crafford, Juergen A. Richt, Robert Swanepoel

**Affiliations:** 1 Vectors and Vector-borne Diseases Research Programme, Department of Veterinary Tropical Diseases, Faculty of Veterinary Science, University of Pretoria, Pretoria, South Africa; 2 Agricultural Research Council-Onderstepoort Veterinary Research Transboundary Animal Diseases Laboratory, Onderstepoort, South Africa; 3 Diagnostic Medicine/Pathobiology, Center of Excellence for Emerging and Zoonotic Animal Diseases (CEEZAD), College of Veterinary Medicine, Kansas State University, Manhattan, Kansas, United States of America

**Keywords:** African swine fever virus, sylvatic circulation, arbovirus, South Africa, extralimital warthogs

## Abstract

African swine fever virus (ASFV) causes a lethal and contagious disease of domestic pigs. In South Africa, the virus historically circulated in warthogs and ornithodorid ticks that were only found in warthog burrows in the north of the country. Regulations implemented in 1935 to prevent transfer of infected animals or products to the south initially proved effective but from 2016 there have been outbreaks of disease in the south that cannot be traced to transfer of infection from the north. From 1963 there were widespread translocations of warthogs to the south, initially from a source considered to be free of ornithodorid ticks. We undertook to determine whether sylvatic circulation of ASFV occurs in the south, including identification of potential new vectors, through testing extralimital warthogs for antibody and ticks for virus. Results of testing warthogs for antibody and other species of ticks for virus will be presented separately. Here we report finding *Ornithodoros* (*Pavlovskyella*) *zumpti* ticks in warthog burrows for the first time. This occurred in the Eastern Cape Province (ECP) in 2019. Since African swine fever was recognised in the ECP for the first time in 2020 and outbreaks of the disease in domestic pigs continue to occur there, priority should be given to determining the distribution range and vector potential of *O.* (*P*.) *zumpti* for ASFV.

The argasid tick *Ornithodoros* (*Pavlovskyella*) *zumpti* was described in 1953 as a new species from the burrow of a four-striped field mouse (*Rhabdomys pumilio* sensu lato) in the Cathcart district of the Eastern Cape Province (ECP) of South Africa (Heisch & Guggisberg 1953). In 1961, the same species was collected approximately 300 km south-west of the type locality from the nest of a Saunders’ vlei rat (*Otomys saundersiae*) in Kariega district, formerly Uitenhage, ECP, during investigation of the bionomics of the tick (Fearnhead 1962). More recently, in 2014, *O.* (*P*.) *zumpti* was again found in Cathcart district, in a porcupine (*Hystrix africaeaustralis*) burrow, and also in the burrow of a springhare (*Pedetes capensis*) in Makhanda district, formerly Grahamstown, during a phylogenetic study of the *Argasidae* (see Figure 1) (Mans et al. 2019).

**FIGURE 1:**
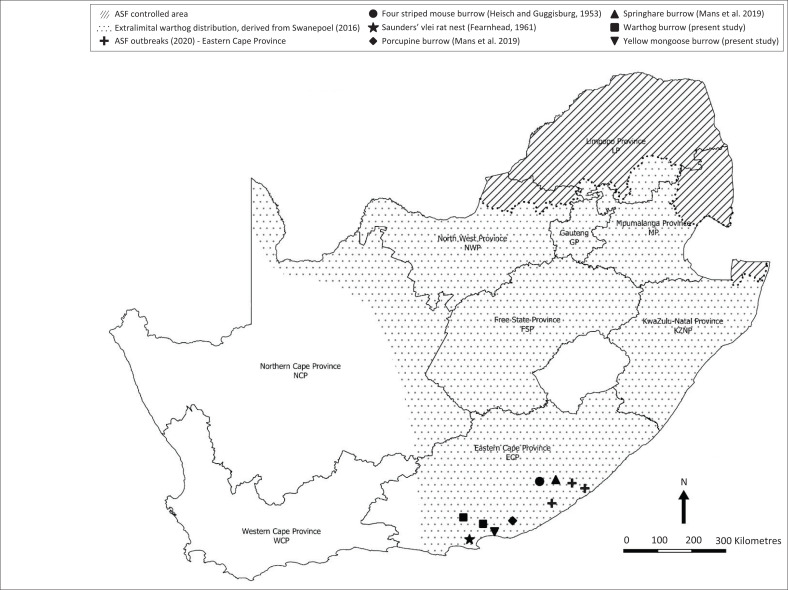
Map showing the African swine fever (ASF) controlled area in South Africa, the distribution of extralimital warthogs derived from Swanepoel (2016), the sites of ASF outbreaks in domestic pigs in the Eastern Cape Province in 2020 and the sites of collection of *Ornithodoros (Pavloskyella) zumpti* ticks from host-associated sites as indicated.

Argasid ticks of the genus *Ornithodoros* serve as biological vectors of African swine fever virus (ASFV) that causes lethal disease in domestic pigs. The virus is maintained in the savannah areas of eastern and southern Africa in sylvatic circulation between warthogs (*Phaecochoerus africanus*) that develop benign viremic infection and eyeless argasid ticks of the *Ornithodoros* (*Ornithodoros*) *moubata* complex that live in warthog burrows (Penrith, Thomson & Bastos 2019). Warthogs develop intense viremia after infection by ticks whilst still confined to their burrows as young piglets, but naïve adults develop viremia of low intensity after infection, and have not been shown to be contagious for domestic pigs. However, they can convey infected ticks to the environs of piggeries or infected warthog offal may be fed to pigs in swill. The infection is contagious in domestic pigs and spreads without need for the tick vectors (Penrith et al. 2019).

Vector-borne transmission of ASFV was first recognised in domestic pigs and *Ornithodoros (Pavlovskyella) erraticus* ticks in Spain after the virus had accidentally been introduced into Europe (Sánchez Botija 1963). Over recent decades, both pig farming and the occurrence of ASFV have spread from east to west across Africa (Penrith et al. 2019) and in 2006 the virus was found in *O.* (*P.*) *sonrai* ticks collected in rodent burrows close to piggeries in Senegal although the epidemiological significance of the finding was regarded as uncertain (Vial et al. 2007). Only members of the subgenus *Ornithodoros* have been identified as vectors of ASFV elsewhere in sub-Saharan Africa. Nevertheless, vector competence studies in various countries abroad have established that the ability to transmit ASFV is a general property of ticks of both the *Ornithodoros* and *Pavlovskyella* subgenera, but the efficiency of transmission varies with virus isolate and species of tick (Pereira De Oliveira et al. 2020).

In South Africa, African swine fever (ASF) was originally prevalent in the northern part of the country where domestic pigs had contact with warthogs (Penrith et al. 2019). Hence, a controlled area was declared in 1935 to include the known distribution range of warthogs at the time (see Figure 1) and regulations were implemented to prevent transfer of infected suids or products to the south (De Kock, Robinson & Keppel 1940). The regulations initially proved effective; but from 2016 onwards there have been a series of ASF outbreaks in domestic pigs south of the controlled area that cannot be linked to transfer of infection from the north. The Department of Agriculture, Land Reform and Rural Development (DALRRD) in 2020, reported the first known outbreaks of ASF in the ECP, and in 2021; the first outbreaks of ASF in the Western Cape Province since the disease was eradicated there in 1939 (DALRRD 2020, 2021; Janse van Rensburg et al. 2020).

From 1963 onwards, there were widespread translocations of warthogs from the north to nature reserves and game ranches in the southern half of South Africa where the extralimital animals flourished to become an invasive species (see Figure 1) (Swanepoel 2016). Originally, the warthogs were obtained by officials of national and provincial parks from Hluhluwe-iMfolozi Game Reserve considered to be free of ornithodorid ticks and ASFV, but it has been suggested that later there were multiple unrecorded translocations from other sources to private nature reserves (Swanepoel 2016). Furthermore, the taxonomy of the Afrotropical members of the subgenus *Ornithodoros* was recently revised, including description of new species (Bakkes et al. 2018). Although information on the distribution ranges and vector capacity of the ticks remains incomplete, the implication is that the potential exists for sylvatic circulation of ASFV in ornithodorids and extralimital warthogs to occur beyond the controlled area in South Africa. We conducted a survey for evidence of ASFV infection in warthogs and ticks outside the controlled area, to be reported separately and in the process encountered new hosts for *O.* (*P.*) *zumpti* ticks.

We collected ornithodorid ticks identified as *O. (P.) zumpti* from warthog burrows in Addo Elephant National Park (Addo ENP), ECP, in 2019 before ASF had been recorded in the province and found no ASFV nucleic acid in the ticks. Approximately 10% weight per volume (w/v) suspensions were prepared by homogenising pools of 1–2 adults or 5–25 nymphal ticks in phosphate-buffered saline, pH 7.2 and automated nucleic acid extraction was performed with IndiMag Pathogen kits (Indical Bioscience, Germany) using slight modifications to the manufacturer’s instruction. A total of 200 µL tick homogenate supernatant was added to 200 µL AL buffer (Qiagen, Germany), incubated at 70 °C for 10 min and 200 µL of the AL lysate added to the extraction buffer. Eluates were tested for ASFV nucleic acid using the real-time quantitative polymerase chain reaction (qPCR) assay of Zsak et al. (2005) as modified by Sunwoo et al. (2019). Positive and no template controls were included in each PCR run.

The ticks were collected in the Nyathi (33.25070S, 24.29166E), Main Camp (33.44492S, 25.78378E) and Colchester (33.41170S, 25.78466E) sections of the park from burrows observed over the course of a week to be occupied by warthogs. The same species was also obtained from a small burrow in Main Camp section (33.44492S, 25.78378E) observed to be occupied by a family of yellow mongooses (*Cynictis penicillata*) (see Figure 1). Identification of the ticks was based on morphology (Heisch & Guggisberg 1953), confirmed by the Gertrud Theiler Tick Museum of the Agricultural Research Council-Onderstepoort Veterinary Research, Onderstepoort, South Africa and by amplification and comparison of partial tick mitochondrial 16s ribosomal ribonucleic acid (rRNA) gene sequences with GenBank data (see Figure 2) (Bakkes et al. 2018; Mans et al. 2019). The *O. (P) zumpti* sequences fell into three clades with 94% – 96% identity and more extensive sequencing studies are required to determine whether the differences represent within-species variation or novel cryptic species.

**FIGURE 2:**
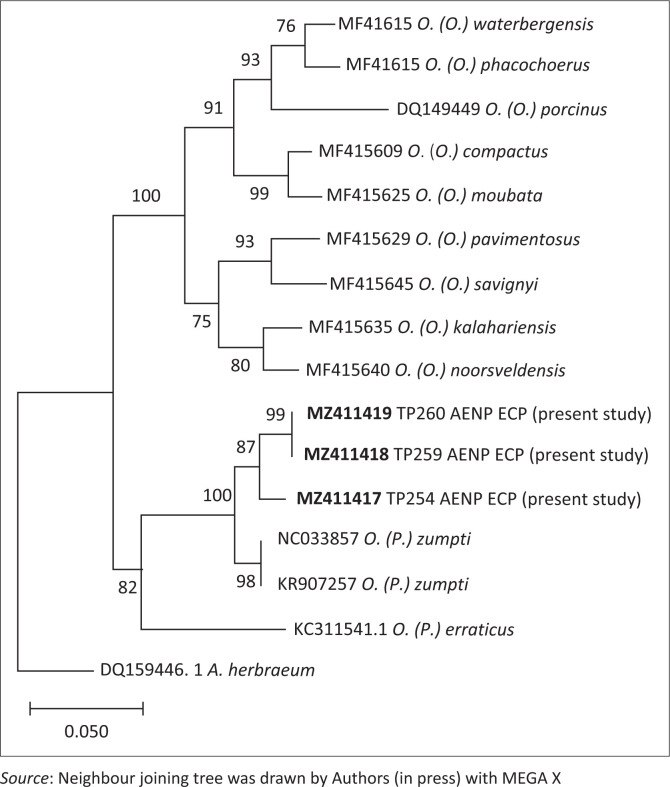
Neighbour-joining tree based on partial mitochondrial 16S rRNA gene sequence (276 nt) depicting phylogenetic relationships between the 3 *Ornithodoros (Pavlovskyella) zumpti* tick pools from the present study (GenBank accession numbers in bold) and representative *Ornithodororos* species sequences from GenBank. The corresponding gene sequence of *Amblyomma hebraeum was included* as an outlier. Percentage bootstrap support values were derived following 10 000 replications. Evolutionary analyses were conducted in MEGA X software.

Partial cytochrome B sequences of mammalian mitochondrial Deoxyribonucleic acid (DNA) were determined from suspensions of partially engorged ticks (Lah et al. 2015) and compared with sequences in GenBank National Center for Biotechnology Information (NCBI) using a BLASTx search to confirm warthogs as blood meal donors for *O. (P.) zumpti*. There was insufficient nucleic acid for cytochrome B analysis from ticks recovered from the mongoose burrow.

The morphology of *O. (P.) zumpti* was well described by Heisch and Guggisberg (1953) but since it is presently the only known representative of the subgenus *Pavlovskyella* in South Africa, preliminary identification can be based on macroscopic features in the field. In each instar of their life cycle, *O. (P.) zumpti* ticks are noticeably smaller than members of the *Ornithodoros* subgenus that occur in warthog burrows. From a dorsal view, ticks of the *Ornithodoros* subgenus have a broadly rounded shape at both the anterior and posterior ends, and are slightly concave bilaterally. *O. (P.) zumpti* ticks have a bluntly pointed hood that covers the basis capituli at the anterior end, whilst the sides are roughly parallel (see Figure 3).

**FIGURE 3:**
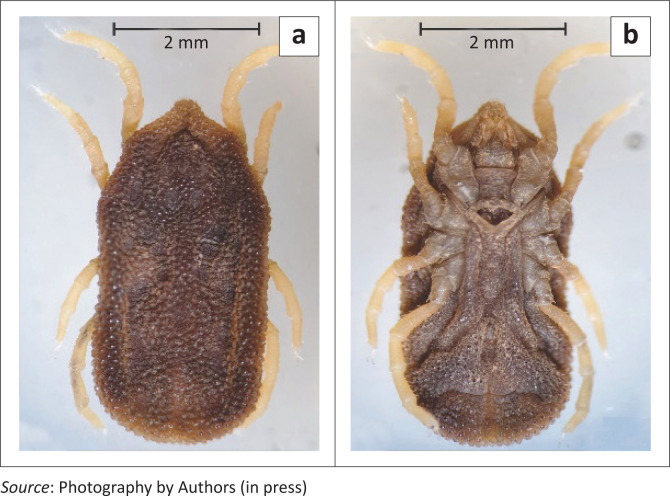
Dorsal and ventral views of adult female *Ornithodoros (Pavlovskyella) zumpti* tick.

In view of the ongoing outbreaks of the ASF in the ECP it should be a priority to determine the distribution range and vector competence of *O.* (*P.*) *zumpti* ticks for ASFV and even to investigate whether warthogs and *O. (P.) zumpti* ticks may already have become involved in circulation of the virus in that province.
